# Exploring implementation of an electronic referral management system and enhanced primary care service for oral surgery: perspectives of patients, providers and practitioners

**DOI:** 10.1186/s12913-018-3424-z

**Published:** 2018-08-20

**Authors:** Joanna Goldthorpe, Caroline Sanders, Richard Macey, Lesley Gough, Jean Rogers, Martin Tickle, Iain Pretty

**Affiliations:** 1 Manchester Centre for Health Psychology, Division of Psychology and Mental Health, University of Manchester, Coupland 1 Building, Manchester, M13 9PL UK; 2 Division of Population Health, Health Services Research & Primary Care, University of Manchester, Williamson Building, Manchester, M13 9PL UK; 3 Division of Dentistry, University of Manchester, J R Moore Building, Manchester, M13 9PL UK; 4 Public Health England, Cheshire and Merseyside PHE Centre 5th Floor, Rail House Lord Nelson Street, Liverpool, L1 1JF UK; 5 NHS England North, Regatta Place, Brunswick Business Park, Summers Road, Liverpool, L3 4BL UK; 6 Colgate Palmolive/ University of Manchester Dental Health Unit, Williams House, Manchester Science Park, Manchester, M15 6SE UK

**Keywords:** Referral management, Primary care, Demand management, Care pathways, Oral surgery, Dentistry, Complex interventions, Qualitative methodology, Implementation

## Abstract

**Background:**

A specialist primary care oral surgery service combined with an electronic referral management and triage system was developed in response to concerns raised around overburdened secondary care services in the UK. Whilst the system has the potential to manage conflicting demand for oral surgery services against an objective need, the new pathway represents a number of challenges to existing working practices and could compromise the sustainability of existing hospital services. The aim of this research was to carry out a qualitative exploration of implementation of a new intervention to gain insight into how these challenges have manifested and been addressed.

**Methods:**

Views were sought from stakeholders (dentists, hospital staff, commissioners and patients) at various time points over 3 years during and after implementation using semi-structured interviews. Normalization Process Theory informed a qualitative thematic analysis which was carried out using data from interview transcripts to identify important emerging issues.

**Results:**

Themes emerging from the data were; amenability to change and assimilation into practice (primary care dentists), compliance and governance, changing perceptions of impact (secondary care staff and commissioners) understanding change in service provision and priorities for treatment (patients). The new pathway impacted stakeholders at different stages of implementation.

**Conclusion:**

Electronic referral management with a primary care advanced service for oral surgery was successfully implemented in a specific area of the UK. The service model evaluated has the potential to be expanded across a wider geographical footprint and to support demand management in other specialist services.

## Background

An advanced primary care oral surgery service combined with an electronic referral management and clinical triage system was developed in response to long hospital waiting lists for tooth extraction in hospital settings within the UK National Health Service (NHS). Primary care dental services act as gatekeepers for secondary care (hospital based) oral surgery, with dental practitioners referring patients to hospital when care is considered too complex for them to safely deliver. This may be due to either surgical complexity or, and in addition to, complex medical histories. Many hospitals fail to meet UK government directives for patients waiting no longer than 18 weeks from referral to treatment (RTT) and the demands of operating in a public healthcare system subject to increasing cuts and efficiency drives was putting pressure on an overburdened service.

Previous research supports the use of referral management and diversion from secondary care to specialist or advanced primary care services to manage demand for less complex procedures, however the context in which new systems are implemented should be considered [15]. For example, a comprehensive review of interventions to manage referrals from General Practitioners to secondary care found evidence that systems involving peer review of referrals improved the quality of referral information, offered specialist contact prior to referral, and facilitated the efficient provision of community-based advanced services. However weaker and conflicting evidence was found for the use of gatekeeping systems (where referrals are rejected and returned if not deemed appropriate) and alterations in remuneration (preventing incentives for referring rather than treating patients) [1]. The electronic referral management system used in this study has some characteristics that could be considered gatekeeping but also incorporated those elements considered best practice by the Kings Fund study [7]. The system, by using a referral form with mandatory clinical fields, the requirement to provide imaging (radiographs) and an electronic submission route has the potential to, via clinical triage, divert referrals to an appropriate provider outside the hospital setting and reject those considered inappropriate. Inappropriate, or unjustified referrals, would typically be considered those where the clinical case for either surgical or medical complexity has not been made, or that the referral is for a clinical service not funded or available (for example, single tooth implants).

A number of studies have looked at the implementation of systems to manage referrals from primary care practitioners. The UK National Health Service (NHS) Electronic Referral System (previously known as “Choose and Book”) [25] is an example of a large, national electronic referral system. Choose and Book has reduced the administrative burden associated with appointment booking and may have reduced the non-attendance rate at hospital clinics [2, 25]. However, this system may not be appropriate for all specialties and in all contexts. Prior to and following the introduction of this UK NHS national system, independent electronic referral management systems were developed with mixed success. For example Maddison et al. [13] evaluated electronic referral management with triage and an adjunct advanced primary care service for uncomplicated musculoskeletal conditions. They found that, although the number of referrals greatly increased following the introduction of referral management, waiting times fell. In addition, duplicate referrals disappeared, and a high degree of patient satisfaction was reported. However Kim et al. [10] found that, although electronic referral management improved access to care there were some barriers to implementation. Some referring clinics reported IT issues, specifically multi step computer login procedures, a lack of computer access and reliable internet connection. Consequently, electronic referrals were reported as taking longer to complete that traditional letter writing (which was often delegated to administrators following dictation), which was associated with lower satisfaction with overall clinical care. This finding highlights the importance of considering the context in which referral management interventions are implemented.

Although precise numbers are unknown, electronic referral management in UK primary care dental practices is not widespread and most practices continue to refer patients via letters written to a named hospital department or consultant. Changing to an electronic system will involve a number of challenges, involving adaptation to different equipment and accompanying skills needed to use equipment effectively. In addition, there may be conceptual changes in ways staff view their roles. For example, the degree of perceived control a dentist has over the patient’s treatment pathway may change (a loss of clinical autonomy) and personal relationships and attachments a dentist may have to a particular organisation or individual within secondary care may become eroded.

When General Dental Practitioners (GDPs) are aware their referrals are being scrutinised, their referral behaviours may change. Studies of peer review interventions, where referral quality has been judged by consultants and fed back to GPs has resulted in some improvement in the quality of referral information and a reduction in the number of overall referrals into secondary care [4, 5, 20], although it may not lead to permanent changes in practice [8]. In addition, shorter waiting times to triage were found for electronic referrals from General Practitioners in primary care medical clinics, when compared with paper referrals [2].

In a key paper relating to demand management, Winpenny et al. [26] carried out a comprehensive, international scoping review of primary care services introduced to address the demand for hospital outpatient services across a number of specialties, including some relating to dentistry. They found that minor surgical procedures could be carried out in primary care safely and effectively, but that provision of such services could stimulate demand by addressing previously unmet need thus impacting on cost-effectiveness. The cost effectiveness of these services was also likely to depend on local context; for example, the price for primary care services versus the secondary care tariff. The Winpenny et al. [26] review included a qualitative study element with individuals implementing such services. The group identified four emerging themes from their interviews: a lack of clarity relating the aims of functions of referral management services (are they around quality improvements, cost savings or patient choice?); challenges of stakeholder adoption and buy-in; practical and administrative difficulties and the impact of perceived effectiveness of the aims and priorities of such services.

In the context of this study, there are a number of perceived advantages to patients attending an advanced service located in primary care: care closer to home, more convenient appointment times and extending opening hours, reduced waiting times (a possible 6 weeks for primary care versus 18 weeks for hospital care [9] and single ‘see and treat’ appointments, rather than separate consultation and treatment appointments reducing costs for patients associated with travel and time off work. Such services are often referred to as Level 2 services, sitting between primary care services (Level 1) and secondary care (Level 3). The commissioning of these services across dental specialities has been advocated within national guidance (NHS [19]).

However, these advantages need to be considered against potential risks using advanced care services. Differences in clinical governance (for example in relation to the reporting of adverse outcomes), combined with the independent nature of oral surgery provision in primary care (often delivered by a single individual) compared to a team approach in hospital *could* threaten the quality of care provided. If patients need to ultimately have treatment in hospital due to complications, or following an assessment visit, savings will be lost, patients experience poorer outcomes and the system will fail [26].

The aim of this study is to explore issues relating to implementation across a 3-year period of referral management in dental practices. It is anticipated that the results may inform the future roll out of this, and similar, interventions across other healthcare settings and footprints and identify issues which may have resonance with the development and evaluation of other referral management and advanced primary care interventions in other contexts and settings.

## Methods

This study was granted approval by NHS Research Ethics Committee (Grampian) Reference 13/NS/0141. All participants provided informed consent prior to taking part in the study and permission was granted for use of anonymous quotes.

### Study context

The implementation phase ran for a 3-year period. It is important to bear in mind the phased stages of implementation covered within this paper, as different stakeholder groups were impacted on to varying degrees across this 3-year time period (see Table 1.)

**Table 1:** Implementation phases

	Year 1	Year 2	Year 3
Study phase	Virtual triage – health needs assessment	Full implementation -Electronic referral management with diversion to specialist primary care service	Full implementation with GDP autonomous decision making – whole system available with option of GDP triage
Participant group involvement & potential impact	Impact on primary care dental practices and commissioning through procurement of specialist primary care services. Consultant triage utilised.	Specialist primary care service begins to offer treatment with some patients treated outside hospital settings; any impact on secondary care will start to be felt by hospital staff.	Autonomous GDP triage available with option remaining to refer to consultants. Commissioning staff look at rolling out the new system across a wider geographical footprint.

The first year (Y1) was used to gather data for the commissioning (via a procurement exercise) of the advanced primary care service. Primary care dental practices referred using the electronic system and consultants triaged referrals through making “virtual” decisions relating to the most appropriate treatment destination, based on referral data. These data were captured and used to inform the commissioning process in Y2. All patients continued to receive their treatment in a hospital setting during this first year, i.e. there was no active diversion of patients, but the potential number, and type, of cases that could be seen in Level 2 services were recorded. The second year (Y2) saw the introduction of a full primary care advanced oral surgery service with consultant triage continuing to determine the ultimate treatment destination of patients. Year 3 (Y3) continued to utilise full electronic referral management with advanced primary care, with general dental practitioners having the option to either perform autonomous decision making regarding the treatment destination of patients, or to continue to defer to consultants for triage.

This paper reports on the qualitative element of a larger mixed methods study, which was carried out at a pilot site with no existing referral management system or primary care oral surgery service in place, allowing the research to capture a number of implementation processes: the commissioning and setting up of a new primary care service, use of a new computerised referral system for primary care dentists, an adjustment of the types and numbers of referrals being received by hospital outpatient departments and any resulting effects on the quality and safety of care received by patients [6].

### Participants

All participants were adults (aged 18 years or older) with the ability to provide informed consent to take part in research. Please see Table 2 for a detailed description of the characteristics of professional participants and Table 3 for a more detailed description of the characteristics of patients who participated in the study.

**Table 2:** Professional participants

Participant number	Role	Practice level of engagement	Organisation type	No of interviews	Focus group yes/ no	Sex (F/M)
CS1	Associate Dentist	High	Primary Care	2	N	F
CS2	Consultant’s secretary	N/A	NHS Trust	1	N	F
CS3	Associate directorate	N/A	NHS Trust	1	N	F
CS4	Practice Manager	High	Primary Care	3	N	F
CS5	Practice Manager	High	Primary Care	1	N	F
CS6	Practice Manager	High	Primary Care	2	N	F
CS7	Principal Dentist	High	Primary Care	2	N	M
CS8	Associate Dentist	High	Primary Care	1	N	F
CS9	Principal Dentist	High	Primary Care	1	N	M
CS10	Principal Dentist	Medium	Primary Care	1	N	M
CS11	Practice Manager	Low	Primary Care	2	N	M
CS12	Community Dentist	Low	Primary Care	1	N	F
CS13	Principal Dentist	High	Primary Care	2	y	M
CS14	Nurse	Medium	Primary Care	2	N	F
CS15	Nurse	Medium	Primary Care	2	N	F
CS16	Administrator	High	Primary Care	1	N	F
CS17	Principal Dentist	High	Primary Care	3	y	F
CS18	Consultant	N/A	NHS Trust	2	y	M
CS19	Deputy Clinical Director	N/A	NHS Trust	1	N	F
CS20	Consultant	N/A	NHS Trust	2	N	M
CS21	Consultant	N/A	NHS Trust	2	y	F
CS22	Commissioner	N/A	PHE	2	N	M
CS21	Commissioner	N/A	PHE	2	N	F
CS24	Consultant	N/A	NHS Trust	1	N	F
CS25	Dentist with special interest	N/A	Primary Care	1	N	M
CS26	Associate dentist	High	Primary Care	1	y	F
CS27	Principal dentist	High	Primary Care	1	N	F
CS28	Principal dentist	High	Primary Care	1	N	M

**Table 3:** Patient participants

Participant ID	Sex	Age	Treatment Setting
1034	F	69	APC
1081	F	43	DGH
1109	F	65	DGH
1138	F	28	DGH
1140	F	61	DGH
1147	F	65	DGH
1151	M	66	DGH
1166	M	42	APC
1172	M	64	DGH
1175	F	51	DGH
1176	F	67	DGH
1189	M	80	DGH
1211	M	69	APC
1213	F	46	APC
1240	M	72	APC
1279	M	57	FTH
1328	M	71	FTH
1403	M	71	DGH
1445	M	64	APC
1921	M	62	DH
1925	F	70	FTH
1928	F	65	DH
1975	F	71	APC
2084	M	51	FTH
2197	M	59	APC
2224	F	80	APC
2324	F	23	DGH
2398	M	58	DH

### Practitioners

Purposive sampling was used to identify key UK NHS staff from participating hospitals, including both clinicians and administrators. Convenience sampling was used to recruit staff from primary care dental practices and the commissioning team including Consultants in Dental Public Health (employed via Public Health England). The study team aimed for representation from the three participating hospitals and from primary care dental practices of various sizes and organisational arrangements (corporate chains and small businesses).

### Primary care

All NHS dental practices operating in the study area, plus the community dental service were approached (*N* = 31) and invited to provide feedback on implementation of the system via email contact. Two practices were excluded; one specialised in orthodontic services and the other referred oral surgery cases to an in-house private service. A total of 28 practices plus the community dental service (*N* = 29) remained. Email invitations to participate in interviews were sent to all practices with a request for the invitation to be circulated amongst their staff. Eighteen staff members responded: seven principal dentists, four practice managers, three associate dentists, two nurses, one administrator and a community dentist. Twelve participants were female and six were male.

### Secondary care

Seven members of staff from the secondary care sites involved in the study responded to email invitations to take part in interviews. Eleven interviews were carried out during years two and three of implementation. Four participants were female and three were male.

### Commissioning team

Two participants were approached directly by telephone and agreed to take part in four interviews carried out in years two and three of implementation. Both participants were female.

### Patients

All participants were UK NHS dental patients who had been referred by their GDP for oral surgery within the geographical area of the study and provided informed consent to take part in one recorded interview. Initially, purposive sampling was used to identify patients of various ages, ethnic backgrounds and postcodes, however as the study progressed it became increasingly clear that the majority of participants had attended two of the four study settings (the advanced primary care service or the district general hospital). Accordingly, our sampling strategy changed with the aim of targeting more participants who had attended the foundation trust and dental hospitals.

Fourteen participants were female and 14 were male. The average age of participants was 60 years and 3 months. Nine participants were treated at the advanced primary care service, four at the foundation trust hospital, three at the dental hospital and 12 participants were treated at the district general hospital.

### Interviews

A total of 29 interviews were carried out with 19 primary care staff over the course of the study (Table 2). We aimed to interview staff from practices with varying levels of engagement with the system within the first 3 months of implementation, then once again at between 6 to 24 months later when new working practices were mores established. Due to some staff leaving and difficulties in accessing other practice members, participants taking part in the repeat interviews forming this phase of the analysis (*N* = 10) were fewer in number than anticipated. Nine interviews were carried out with seven secondary care staff and two commissioners took part in two interviews each. All practitioner interviews were carried out either over the telephone or face to face at the participant’s place of work. Thirty patients took part in telephone interviews carried out over the telephone. One recording proved to be intelligible and the recorder failed one interview, leaving a total of 28 interview transcriptions in the analysis. All interviews typically lasted 30–45 min. The interview schedule was informed by constructs from Normalization Process Theory (NPT) [14].

### Data analysis

The aim of the grounded theory approach is to generate new theory or expand existing theory through an in-depth exploration of the data. Although the analysis explored lived events as described by participants rather than generated theory, techniques used in a grounded theory approach can be transferred to thematic analysis to ensure a deductive approach is taken (that findings are grounded in the data rather than in researchers’ preconceptions). Stages of coding comprised initial coding of text segments, followed by re-coding and memo writing to generate conceptual themes. Themes were compared within and across cases, paying attention to negative cases and possible reasons for differences. The generation of themes was primarily inductive in that themes were grounded in the initial coding of the data, however once themes were identified they were mapped deductively to existing core constructs from Normalisation Process Theory (NPT) [14]. NPT is a theory of organisational change, developed to study implementation, facilitators and barriers of new work practices and innovations within health systems. NPT offers a flexible and pragmatic approach that can help us understand factors that affect routine incorporation of complex interventions into everyday practice. Moreover, the application of NPT in the analysis of qualitative data allows for the extraction of a more abstract level of ideas for implementation of complex interventions, which could apply to other contexts and research settings [17].

Data were organised with the aid of qualitative data software package QSR NVivo (QSR International, London, UK). An audit trail of all stages of the analysis to maximise credibility, dependability, confirmability and transferability [12, 16] was created through sharing and saving of NVIVO files at various stages of the analysis, recording group decision making and archiving emails relating to the analytic process. Four of the authors (JG, CS, RM & IP) discussed emerging themes to enable refinement of conceptual categories and to discuss common threads or differences across the different respondent groups to ensure reliability. Any discordance in the identification of themes and codes was resolved through discussion. Further trustworthiness of findings was established through triangulating findings, study documents and quantitative data (please see Goldthorpe et al. [6] for a full description of other data sources collected).

## Results

Please see Table 4 for a summary of the thematic analysis in relation to each group of participants, and how this maps on to the NPT constructs.

**Table 4:** Summary of thematic analysis for each group of participants in relation to NPT constructs

NPT Construct	Coherence	Cognitive Participation	Collective Action	Reflexive Monitoring
Primary Care Practitioners			Amenability to change	Assimilation into practice
Secondary care practitioners and commissioning team		Compliance and governance	Compliance and governance	Changing perceptions of impact
Patients	Understanding change in service provision			Priorities for treatment

### Primary care

The first 3 months Y1 (virtual) implementation proved to be a vital stage for overcoming barriers associated with equipment and IT skills of staff. Dental practices need a broadband connection and a least one computer connected to the internet. Users enter their user name and password, followed by an additional level of authentication, which involved entering a PIN number, unique to each login. Skills required to operate the system are analogous to those used when making a typical online purchase or booking. Training sessions were held at introduction meetings and practices were offered site visits from a member of the research team for one to one training.

### Amenability to change

Amenability to change relates to the readiness and enthusiasm for change, which motivated staff to engage with the new system. This links to the NPT construct of Collective Action, which describes the practical tasks people need to complete in order to operationalize a new practice. Within this construct, the Interactional Workability component describes the practical interaction with the instruments or technologies of new interventions. This has resonance with issues arising in relation to the new technology associated with the referral management system.

Having the appropriate infrastructure and skill set already in place was hugely advantageous and facilitated successful implementation. Overall, staff working in practices that had already invested in up to date technology found the electronic referral management system had low impact on their existing working practices, and accepted the new way of referring;

*There were no real changes needed, other than an enthusiasm for change, which we had* (CS9, Principal Dentist).

Some staff were reluctant to acquire new skills needed to operate the system; colleagues described them as being “technophobes”, individuals who had not yet developed skills around use of technology, or did not habitually use electronic communication systems;

*“You know, they find it hard enough to check their emails, let alone manage a system like this”* (CS10, Principal Dentist).

Older staff were cited as a group reluctant to embrace electronic referral management. This is supported by responses from some older staff members, who themselves expressed reluctance to move away from letter writing and the postal service when referring patients. When individuals were unused to engaging with technology outside the work setting, moving to an electronic system specifically for referrals was a low priority within a larger set of work-based tasks, particularly in staff members with imminent plans for retirement;

*“If I was staying longer, I would get used to computers, but I’m going to retire anyway, it doesn’t seem worth it” (CS15 Practice Nurse).*

It was the elusive “enthusiasm for change”, as cited by a previous respondent that emerged as a key facilitator to implementation, when changes to infrastructure and skills set were needed. Many practices were willing to make investments in time and resources, facilitating adaptation to electronic referring. This could mean staff training or investment in up to date software and in a small number of cases, purchasing a computer. In addition, some (mainly smaller) practices managed to effectively navigate reluctance to acquire new skills by delegating oral surgery referrals to computer - literate individuals;

*I’ve crossed off half an hour every week to go through them with him … because I input all the data, so I can input the date, address, he doesn’t have to sit through that boring bit … just the clinical. Even the medical history I do, but I take him [dentist] through all that to make sure it is accurate, but I input all that … it’s 5 min for one.* (CS4, Practice Manager).

Conversely, a small number of practices featured management or key staff low in amenability to change, which proved to be a particularly recalcitrant barrier to implementation. For example, one practice would not engage with the new system or the research team to any extent. Another practice, which was part of a corporate dental care chain, employed a manager who had administrative responsibilities but no clinical background. In this case, the manager did not seem to have the remit to influence change within staff, despite best attempts to implement the new system at his practice;

*I have to say I have tried but the online referral system has failed to take off at the practice. Dentists have tried but still preferred paper referrals as they didn’t like the password process or the need to cut and paste details* (CS11 Practice Manager).

### Assimilation into practice

Assimilation into practice describes the process of demonstrative feedback, both through system use contributing to the management of workloads, as well as verbal feedback from patients attending the advanced primary care service. Reflexive Monitoring is a relevant NPT construct that refers to the appraisal work participants carry out in order to evaluate the ways they are affected by new systems and interventions. Communal Appraisal and Individual Appraisal are two components of Reflexive Monitoring that refer to the ways in which individuals and groups decide how a new system is working for them.

Practices who engaged with the system during the first months of the implementation phases continued to use the system throughout the study. This enabled their patients to access the advanced primary care system in Y2 and Y3 of implementation (referral into the service could only be made using the online referral management system). Benefits to patients and practitioners were demonstrated as time passed, which aided the process of assimilation into normal practice through demonstrative positive feedback. Patients were perceived to be receiving appropriate treatment, and feedback did not provide reason for concerns around care quality and patient safety to arise. The main benefits cited by practice staff were associated with efficiency of the system to deliver referrals to the appropriate destination immediately, bypassing the slower traditional mailing system. Similarly, referrals could be chased immediately, online without the need to write to or telephone clinics;

*The main benefit is that you can see where the referral is up to… in the past we have sent letters that have got lost in the post and then you don’t realise until the patient comes back and say they still haven’t heard anything. With this system, you’ve got a reference number and you know that it’s been received … we actually had one [patient] the other day we could tell her she’d gone straight to hospital so the referral is sitting there (Practice Manager, CS6).*

Patients generally provided positive feedback to practice staff regarding the advanced primary care service. The shorter waiting time for appointments and ability to consult and treat at the same appointment was aligned to patient priorities to be treated as quickly as possible. The geographical location of advanced primary care clinics had been chosen specifically to serve both the North and South of the local area and ease of public transport and parking was considered. Convenience of the clinics, along with the opportunity to access evening or weekend appointments was also reported as popular with patients;

*Some of them that go there are delighted because they’ve been seen, he had extra clinics on a Saturday and it was just down the road (CS13, Principal Dentist).*

A practice manager however, described how a patient had stated a preference to be treated at the Dental Hospital, as she could reach it easily by train, despite the primary care service operating from a closer surgery. However, the practitioner felt this was an idiosyncratic preference brought about by habitually making the journey to the city centre, rather than typical of a widespread aversion to being treated outside a hospital setting:

*People are used to going into town and back and then that’s it, they don’t really venture much further … she’s been there, she knows where she’s going even though [the primary care service] is closer than town (Practice manager, CS4).*

After the second year of implementation with full diversion to the advanced primary care service, dentists were given the option to bypass the consultant triage element of the system, allowing them to take autonomous control of the treatment destination of patients. Although autonomous decision-making was found to be mostly acceptable to GDPs, participants in this study did not express a strong preference for either method of patient triage. Views suggesting closer relationship with patients were more suited to the task of deciding where their patients were treated were not expressed, and it was not felt important to tell patients where they would be treated at point of referral.

*I mean, if you found the [consultant-led] system works well, if that couldn’t be our choice, it wouldn’t unduly bother me to say [to the patient], “you’ll find out in a week where you’re going*” (F2 associate dentist).

Furthermore, autonomous decision making had not been assimilated into the normal working practice of some dentists, who continued to defer triage to consultants throughout the final year of implementation. This may be because consultant led triage appeared to offer a layer of additional expertise, which could possibly add to the quality of the overall service. For example, this GDP preferred to defer decision making for cases that proved difficult to evaluate;

*Sometimes I’m a bit unsure of whether it should be done in a hospital setting or not … I’ve just put in for it to be triaged by the team* (CS17, principal dentist).

### Secondary care practitioners and commissioning team

Shared priorities of secondary care staff and the commissioning team were reflected in the data provided by participants working in both types of organisations, therefore analysis was combined.

### Compliance and governance

The first theme emerging form this merged data was compliance and governance, which relates to concerns held by consultants regarding upholding clinical standards and supervision in the context of the new primary care service and changing perceptions of impact, which describe perceived threats to secondary care services throughout and beyond the study period. This theme relates to the NPT constructs of cognitive participation and collective action. Cognitive participation describes the relational work individuals and groups of people do to build an association with a new technology or complex intervention and this describes the process of understanding a more specific arrangement relating at clinical governance was needed in relation to the new primary care service. Collective action describes how practices are enacted and put into operation and this relates to the work carried out in order to put these structures in place in response to an identified need.

The introduction of the advanced primary care service meant that hospital-based consultants conceded control of the care of some oral surgery patients, as the new service operated outside the hospital setting, and was not directly overseen by a consultant. It was felt by senior NHS staff, such as commissioners that this change was necessary and appropriate due to the backdrop of pressures on resources caused by increasing numbers of numbers of patients referred for minor oral surgery procedures, and changes in skill mix were seen as inevitable;

*“The time has come really that we can’t afford for maxillofacial surgeons to be doing tooth extractions” (Commissioner, CS23).*

Participants had concerns around two-way communications between dental practices and secondary care staff. The nature of communication between the two specialties had been altered by electronic referral management and keeping lines of communication open to discuss clinical issues was important to consultants. It was felt that some clinical issues needed to be re-visited and a consensus established around guidelines relating to referrals from primary care. For example one consultant had particular concerns relating to specific medical histories important to oral surgery procedures, such as the increased number of patients taking bisphosphonates who were at particular risk of extensive bleeding during surgery.

*Overall it’s a good system but that is the thing that concerns me, you know, because traditionally dentists would just pick up the phone to us and say, “oh, I’m worried about such and such what should I do” and you go, “alright just send it up”, but if they have to send it through that process now I’m then going to be potentially contradicted … it may be that when they triage them, anybody who is on bisphosphonates, they agree that they should be done in the hospital, “I don’t know, but I’m not sure what the guidelines are” (Consultant, District General CS20).*

In addition to concerns around patient care, some consultants were concerned with ensuring that the primary care service providers were protected. The advanced service was led by a primary care practitioner with enhanced skills in oral surgery and although clinical staff understood the procurement process ensured levels of compliance and clinical safety were adhered to, some concerns around clinical supervision and protection against litigation were raised.

*Most GDPs run businesses and the last thing they want is things going wrong in the business … we’ve got a litigation department who’ll deal with it and yes, it’s happened but we’re ok, we will carry on. But if somebody walks out [of APC service] and says, “I got butchered by the doctor” it doesn’t take long for your business to be fairly well wrecked (Consultant, Dental Hospital CS18).*

In response to these concerns, a number of meetings were held to discuss clinical issues and to agree on agreed procedures for communication between primary care and consultants. In addition, a single named consultant was appointed as clinical supervisor for the advanced primary care service. These discussions and actions were able to assuage the concerns of consultants, and contributed towards developing an improved procurement protocol for future service delivery;

*I think some lessons learnt there about being absolutely clear who is providing that service, and if it is a dentist with enhanced skill … that we have them linked into consultants or specialists for clinical supervision. (CS22 Commissioning team).*

### Changing perceptions of impact

This theme relates to the perceived impact of the new service on existing services, and the contrasting views around whether this impact was realised. The NPT construct of reflexive monitoring relates to the appraisal work people do in relation to the introduction of new processes. All four components of Reflexive Monitoring are relevant to these findings. Systemisation describes the processes involved in determining how effective and useful changes are; Individual Appraisal and Communal Appraisal relates to experiential experiences that help to determine the worth of the intervention; Reconfiguration is appraisal work that may help to redefine or alter practices or components of a new intervention.

One aim of diverting less complex cases to advanced primary care services was to bring about a reduction in the number patients treated in hospital outpatient clinics resulting in benefits such as: shorter waiting lists for patients, reduced pressure on trusts, more manageable workload for staff and an end to waiting list initiatives, such as Saturday clinics.

*“I think it will improve waiting times more than anything else .. I think that [referral management] will bring back the number of patients that we need to see to a reasonable level so we don’t need to run additional sessions … bring it back to something that’s manageable” (Consultant with experience of all hospitals CS24).*

Additional fears were expressed by secondary care staff at the start of the study around reduction in patient numbers causing the destabilisation of existing services, threats to existing staff positions and a reduction in cases suitable for training purposes impacting on junior staff and students. A change in the mix of complexity of cases being undertaken was of particular concern to the Dental Hospital, where commitments to train undergraduate dental students needed to be met. Data generated by the referral process indicated the largest impact would be felt by the District General Hospital, however views from staff at this hospital were inconsistent across the study period. During the early months of the full implementation period (Y2) staff from this hospital shared concerns about loss of lower grade surgical staff and that newly qualified dental nurses would have reduced exposure to simple procedures, narrowing their training experiences.

*Clinics are getting smaller, nurses are falling on their training, that’s the only thing I can tell you from what I’ve seen … I would say probably in the last 3 months (Deputy clinical director, District General Hospital CS19).*

However, fears and assumptions around the introduction of referral management were not generally borne out by experience over time. This may have been due to hospitals receiving referrals from a much wider geographical area than that included in the parameters of the research, however as time went on less of an impact was also perceived by staff at the District General Hospital, where the reduction in less complex cases was demonstrated by quantitative data to be higher;

*I was expecting [the reduction] to be about 30 or 40%, but I’m still seeing loads … its just my gut feeling and the figures will tell but I see some of them and I think, “well that is a level 2”[suitable for the advanced primary care service], but it’s still coming to us (Consultant, CS20).*

Fears regarding potential future impact on teaching cases were also assuaged over time, as consultants became aware that algorithms within the referral management system could be created to retain and divert a certain number of simpler oral surgery cases into hospitals, based on need. For this work in the long term, it was assumed that a formal needs assessment would need to take place within trusts and a formal plan generated. In addition, it was felt that independence of the practitioner undertaking the triage process would need to be maintained.

*So long as you are keeping separate the people that are triaging from the end user in effect, then that’s ok (Consultant with experience of all hospitals CS24).*

Many concerns surrounding the impact of referral management for oral surgery expressed at the start of the research period were alleviated over the period if this research. However, commissioning plans to roll out the intervention across a wider geographical footprint have resulted in the reintroduction of some of the potential threats expressed early in the study. In particular, concerns relating to loss of extra staff recruited to make waiting list targets and destabilisation of services in general remain as more dentists will be encouraged to engage with electronic referral management and more specialist or advanced primary care services are commissioned.

*The concerns we’ve got in the long term is that if the information from this project ends up being extrapolated across the whole region, we may find there’s a huge effect … although the numbers involved are too small to make any difference to us at the moment (Consultant, Dental Hospital, CS18).*

### Patients

#### Understanding change in service provision

The NPT construct of Coherence refers to the ways in which participants make sense of operationalising new ways of working and levels of understanding in relation to the philosophy and aims of new practices. Two components of coherence, communal and individual specification relate to how both individuals and groups understand what is required of them in order to fit into a new system. In this case, group specification relates to the ways in which dental organisations communicated the changes in treatments pathway to patients, and how patients made sense of the information available to them to access their treatment.

Patients generally had a good general understanding of the clinical reasons for their treatment and why they were being referred. They reported GDPs offering good explanations of procedures referred for and any reasons for interim treatments such as antibiotics. Participants were satisfied overall with treatment and levels of clinical safety and expertise in both primary and secondary care and there was a sense of indifference relating to which service carried out the treatment. For example, the following participant had experience of treatment in both hospital and advanced primary care settings and found them as equally acceptable as treatment at his local dental practice:

*[Advanced primary care service] was very similar to the [hospital] experience … he actually told me that the reason I had been referred [to advanced primary care] was because of the angle of the root or something. But on both occasions they seemed to take the tooth out very easily. Both times I have walked away and gone, “why wasn’t that done at the dentist?”* (1166, Male, 42, Advanced primary care).

A specific preference for primary or secondary care was stated only in a small number of case for idiosyncratic reasons which were not related to intrinsic elements of the service (e.g. “I just don’t like hospitals”), or for patients who had previous experience of oral surgery in hospital under general anaesthetic (not offered outside the hospital clinics). The following patient was typical in being able to accept this change, providing she had a suitable alternative to manage her anxiety;

*“The only thing that frightened me was that it was done under a local anaesthetic where the other one had been done under a general. I just got the impression it was better for the patient in some respects, you’re not under [general] anaesthetic as it can be a bit dodgy sometimes can’t it? …I had a couple of Valium from my doctor instead” (1147, female, 65, District General Hospital).*

As patients had little understanding of changes in treatment pathways or provision, attempts to chase treatment appointments often caused confusion as incorrect assumptions could be made about where treatment would take place. Often staff in general dental practices were unsure how to look up triage decisions and ultimate referral destinations on the system, which exacerbated frustrating attempts to chase appointments.

*“The worst thing was having to wait for the appointment and then having to chase everything myself. I seemed to be coming up against brick walls all the time. And the fact that the dentists didn’t know, the receptionist didn’t know where I should be having it … the right hand didn’t seem to know what the left hand was doing*” (1140, female, 61, District general hospital).

### Priorities for treatment

This theme relates to the individual appraisal component of the NPT Reflexive Monitoring construct. Patients appraised their levels of satisfaction with the service they attended in relation to expectations based on previous dental or hospital outpatient experiences.

Patients generally did not prefer any one of the treatment settings on offer and findings relating to preferences and priorities were similar for participants attending services at all sites included in the study. A short waiting time was reported as particularly important to participants, both in terms of time from referral to appointment and time to see a clinician at the treatment setting. Most frustration was expressed specifically by those in pain or discomfort, who may have to wait for up to 16 weeks for a consultation appointment. These patients in particular appreciated the shorter waiting times offered by the primary care service;

*“I was happy to just have it dealt with because it was really beginning to bug me … If it was giving me as much pain I’d be quite happy to go back to [advanced primary care service] rather than wait 2 months to be seen [in hospital] …”* (1975, female, 71, advanced primary care).

Waiting for appointments was more acceptable to participants with less debilitating symptoms and when expectations were managed. Periods of time spent waiting for an appointment could be acceptable, provided they knew approximately how long they would be waiting and the reasons behind the wait.

*Just having an idea of what is expected and what is reasonable … helps. Everyone’s concern ultimately is, “have they forgotten about me? I only got an appointment a couple of days ago when I phoned up to see what happened”… you being to think, “maybe there has been a little mistake*” (1211, M, 69, Advanced primary care).

Participants highly valued good, patient centred care. Interpersonal skills of health care professionals were important, particularly to anxious patients. Participants appreciated having their anxiety acknowledged and accepted by staff and described distraction techniques, such as making jokes and general conversation as effective in putting them at ease.

“*To be honest, I was quite surprised that I actually wasn’t nervous. They all made me feel dead comfortable. There were two nurses and a surgeon and they were all having a bit of a laugh and a joke, to put me at ease. So, I thought that was nice, that they weren’t just sitting there in silence*” (1138, female, 28, District general hospital).

Participants (again, particularly anxious patients) liked to have a good amount of information around what to expect at their appointments for consultation and treatments, and to have the opportunity to ask questions in a non – judgemental environment. This could include familiarity with staff and their roles, in addition to the surgery itself;

*“I couldn’t work out what the dental nurses were doing … I need to know what people are doing… The clinician was very good, he explained everything and every question that I asked, he didn’t look at me like I was stupid” (*1140, female, 61, District General Hospital).

## Discussion

For primary care dental practices, early engagement with the electronic referral system was vital to sustained use of the system and for this to take place amenability to change needed to exist within staff who had the authority and capability to implement change and overcome barriers such as lack of IT skills and infrastructure. Over time, the study demonstrated that use of the system could successfully become embedded into everyday practice. This process was encouraged through feedback to referring practice from patients attending the advanced primary care service and through increasing familiarity with the online referral system. More interviews were carried out with primary care practitioners during early implementation rather than in years two and three post-implementation and it may be that findings relating to this group of participants are more relevant to the early stages of implementing and adapting to the new referral management system.

Following procurement of the advanced primary care service during the second year of implementation, some secondary care hospital-based consultants raised issues relating to compliance and governance. In response, meetings were held with three hospital consultants, the advanced primary care practitioner and the commissioning team which resulted in a number of procedures being developed, such as guidelines around referring patients using Bisphosphonates (increased risk of osteonecrosis following dental extraction), and revision of clinical governance arrangements for the advanced primary care practitioner. Concerns voiced by secondary care oral surgery services relating to loss of capacity and a reduction in appropriate teaching cases for junior staff were not realised during the study period. Similar fears do however remain regarding the impact of further implementation of electronic referral management and particularly, the procurement of more advanced primary care providers to operate across a wider local geographical area. Thus, perceived effects on hospital oral surgery services not realised within this pilot study may still materialise following roll out of the intervention and consideration needs to be given to organisation and provision of teaching and training of junior surgical staff in the long term.

Patients were mostly unaware of changes to their local oral surgery service, unless they had cause to query a delayed appointment. Their priorities for treatment were expedience, convenience and quality of care, rather than care provided in a particular setting. For patients with anxieties around dental treatment the wider choice of anaesthetic available in hospitals (sedation and general aesthetic) may make referral to secondary care more desirable, however the much shorter waiting times on offer for appointments in the advanced primary care service may override this element, particularly for those with painful symptoms.

Overall, the findings of this study support those of other authors exploring electronic referral management and new primary care services for less complex procedures. Kim et al. [10] reported that patients were satisfied with access to care following implementation of electronic referral management, however some barriers to implementation related to IT systems and a perception that referrals took longer to complete. In our study, GDPs were sometimes reluctant to engage with the electronic system, mostly due to IT related issues and resistance to change. These practitioners tended to resume use of paper referrals, negating the electronic referral management system and thus negating the referral pathway to the advanced primary care service. This resulted in access to the primary care service (with potential shorter waiting lists) being blocked for some patients. Winpenny [26] similarly found a lack of coherence relating to the aims and mechanisms of systems designed to reduce a burden on secondary care services across specialties, stakeholder challenges relating to adoption of new ways of working and perceived effectiveness of service reorganisation.

The overall acceptance of changes to the oral surgery service by the majority of primary and secondary care practitioners in this study is supported by other research which has found that in the modern healthcare workforce, organisational changes tend to be consensual rather than combative [18]. Although not examined in this particular study, the introduction of change within the organisation of oral surgery services raises a number of issues regarding the changing nature and boundaries of traditional professional roles in dentistry, which could be explored in future research. For example, the introduction of new technology into dental practices may highlight a deficit in the administrative skill set of very experienced and established staff members. Furthermore, secondary care staff may find themselves dealing with a more intense and complex workload requiring specific specialist skills, due to the diversion of simpler cases into primary care services. A review of contested professional role boundaries in the health sector found that the direction of boundary crossing (virtual or horizontal) may influence acceptability of changing roles in addition to perceptions of those in established roles [3]. However, when changes are underpinned by wider policy and context (as in this case of referral management for oral surgery) changes in professional boundaries can be negated and accepted [22].

A number of research studies focusing on dental patients have found the management of pain and anxiety greatly influences their perceptions of service quality [24] and furthermore lack of attendance at appointments is largely due to anxiety around dental treatments [11, 23]. It may be that our sample of patients reflected those with moderate to low dental anxiety, who attended their appointments and thus were able to provide data relating to their experience. A targeted study of patients with high levels of dental anxiety may reveal that treatment in secondary care is significantly favoured over advanced primary care due to the greater availability of sedation and general anaesthetic. This may also affect the context of the primary care dental consultation, with anxious patients expressing a firm preference for secondary care and exerting influence on the referral behaviours of dentists.

Furthermore, patients may have been influenced by positive interpersonal relationships with staff providing a particular service, causing impressions of the service in which they work to become skewed. This is known as the “halo effect” [21]. The issues raised above were outside the scope of this study, and thus warrant further exploration.

There were a limited number of secondary care practitioners who were suitable for recruitment to this study. Many of those approached felt they did not have sufficient awareness of the new referral management system or advanced primary care service to contribute to data collection in a meaningful way. It may be that when the service is introduced on a wider scale and any impact becomes more apparent, opportunities to explore the views of a larger cohort of hospital staff may arise. An additional limitation of the study is that it focuses on one medical speciality, in one geographical area and the findings may not be generalizable to other settings (specialties and localities). Further research is needed to investigate if new primary care services set up to manage high demand for secondary care outpatient services in other geographical areas, under different geopolitical contexts and for different specialties yield similar results.

## Conclusion

This study has found that electronic referral management with a primary care advanced service for oral surgery can be successfully implemented in a specific area of the UK and has the potential to be rolled out across a wider geographical footprint and to support demand management in other advanced services. Patients appreciated the convenience and shorter waiting times offered by the primary care oral surgery service and quality of care was considered to be at similar levels for both primary and secondary care services. The electronic referral management system had greatest impact on primary care dental practices. Despite the mandatory nature of the change to an electronic referral management system there was room for individuals to negate the system and return to paper and post referrals, with the result that some patients were not able to access the primary care service. For implementation to be successful, sufficient IT infrastructure needs to be in place and key staff members should take a lead on supporting change within the practice teams. Anticipated destabilising effects on hospital services were not realised during the course of this study, however concerns around the potential for wider implementation of the new system to impact on the current workforce and reduce training opportunities for junior staff remain.

## Abbreviations

APC: Advanced Primary Care
DGH: District General Hospital
DH: Dental Hospital
DwSpI: Dentist with Special Interest
FTH: Foundation Trust Hospital
GDP: General Dental Practitioner (Primary Care)
NHS: National Health Service (United Kingdom)
NPT: Normalization Process Theory
